# Cases Illustrative of the Pathology and Surgical Treatment of Some of the Diseases of the Testicle; with Observations

**Published:** 1827-10

**Authors:** B. C. Brodie


### DISEASES OF THE TESTICLE.

*Cases illustrative of the Pathology and Surgical Treatment of some of the Diseases of the Testicle; with Observations.* By B. C. Brodie, F.R.S. &c.

(Continued from vol. i. p. 528.)

### *Hæmatocele.*

Systematic writers have applied the term Hæmatocele, first, to those extravasations of blood which take place within the tunica vaginalis; secondly, to extravasations into the cellular texture of the scrotum; thirdly, to extravasations into the cellular texture surrounding the spermatic chord.

But these cases are essentially different from each other. The two latter correspond to common ecchymosis, and the former alone can be regarded as peculiar to the testicle, or its appendages. In this paper the term is employed in the first sense only,—that is, as signifying an extravasation of blood into the tunica vaginalis.

1. Hæmatocele may take place where the parts are previously free from disease; a blood-vessel being ruptured either in consequence of a blow or strain; or spontaneously, that is, without any evident cause. Under these circumstances, if the extravasation has taken place only to a small extent, it may, if left to itself, in the course of some months, or after the lapse of one or two years, become gradually absorbed. If, however, the extravasation be very considerable, such complete absorption does not take place, and more or less of the tumor remains ever afterwards, or at any rate for several years.

Case XXVII. *Hæmatocele from accidental Injury, in which no Operation was performed, and only a partial Absorption of the Tumor took place.*

H. K—, about thirty years of age, on the 20th of September, 1820, received a blow on one testicle. The testicle immediately became swollen, and the swelling very soon was equal in size to a goose's egg. I saw him a few days afterwards, in consultation with another medical gentleman, and we recommended the application of spirituous lotion, and that he should remain in a state of rest. Sometime afterwards we prescribed friction with mercurial ointment and camphor. The tumor, however, remained unaltered for upwards of a year; at the end of which time it began spontaneously to diminish in size. The diminution proceeded slowly until about two-thirds of the tumor had disappeared, and then ceased. From the beginning of the year 1825 to August 1826, there was (as I am informed) little or no alteration in it. At this last period, the testicle was to be felt in a natural state, and the tumor was perceived as a hard mass adjoining the testicle, but not confounded with it; whereas formerly the tumor and the testicle were not to be distinguished from each other.

Here, although a considerable part of the extravasated blood remained at the end of six years, there is no reason to believe that this was attended with any injurious consequences to the testicle itself. The following case, however, would lead us to suspect that a large hæmatocele cannot be allowed to remain, in all instances, with the same impunity.

Case XXVIII. *Case in which there was reason to believe that the Patient had laboured under an Hæmatocele of long standing, and in which the glandular Structure of the Testicle had disappeared, probably in consequence of the long-continued Pressure of the Extravasation.*

In examining the body of an elderly negro, who died in St. George's Hospital of a disease in his lungs, and in whom also the arteries were found to be extensively ossified, I observed a large tumor on the right side of the scrotum, which had the shape and appearance of a hydrocele, and which gave to the touch a distinct feeling of fluctuation. On further examination, it was found that the tumor was formed by the tunica vaginalis distended with about twelve ounces of fluid, having the appearance of coffee-grounds, with numerous masses of solid substance, manifestly fragments of coagulum, floating in it. The tunica vaginalis itself was much thickened. The substance of the testicle, the tunica albuginea, and the tunica vaginalis immediately covering it, were entirely destroyed; so that not a vestige of these parts could be discovered. The vas deferens adhered to the posterior part of the tumor, and was imperceptibly lost at the part where it usually joins the testicle.

Another case, exactly corresponding to the one just described, has lately fallen under my observation. The appearances on dissection were precisely similar; but here also I unfortunately lost an opportunity of learning the history of the case during the patient's life-time.

Cases of this variety of hæmatocele, in which, from the large size of the tumor, there is reason to believe that the extravasation is not likely to become absorbed, may be treated by making a simple puncture with a lancet. The four following cases are selected as illustrating the various effects produced by this method of treatment.

Case XXIX. *Hæmatocele occurring spontaneously, in which the Tumor having been punctured by a Lancet, Inflammation of the Tunica Vaginalis took place, followed by Suppuration, and the Patient was cured.*

W. Cook, nineteen years of age, admitted into St. George's Hospital on the 27th of December, 1815. Seven months before his admission, he observed a swelling in the situation of the right testicle. The swelling took place without any evident cause, and suddenly, so that in the course of some hours the testicle appeared to be of twelve times its natural bulk. There was little or no pain. After this the swelling remained unaltered, until about five weeks previous to the man's admission into the hospital, when it was punctured by a surgeon whom he consulted. Some bloody fluid was evacuated, but the swelling did not wholly subside, and it again increased after the puncture.

At the time of the patient coming to the hospital, there was a tumor in the situation of the testicle, of ten or twelve times the size of the latter. The fluctuation of fluid was distinct at the upper and anterior part; the rest of the tumor was evidently composed of solid substance.

December 29th, a puncture was madewith a lancet, and about four ounces of blood y fluid wer e evacuated. A considerable quantity of solid substance remained after the fluid was drawn off. The puncture healed by the first intention.

Inflammation followed the operation. Fluid was again collected within the tunica vaginalis. On the 10th of January, 1816, an abscess burst, discharging pus, with portions of half-dissolved coagulum floating in it.

The abscess continued to discharge matter with portions of half-dissolved coagulum; and, as this discharge continued, the bulk of the solid tumor became diminished.

In the course of a few weeks the discharge ceased, the abscess healed, and the patient was discharged, with little or no swelling left.

Case XXX. *Hæmatocele, in which Inflammation and Suppuration followed the Puncture of the Tunica Vaginalis, and the Patient was cured.*

James Roles, sixty years of age, admitted into St. George's Hospital, October 1st, 1817. A month before his admission, he received a blow on his left testicle. A swelling immediately took place, which gradually increased for some hours, and afterwards remained, with little or no pain, and without undergoing any sensible alteration, up to the day of the man being admitted into the hospital.

At this time there was a swelling, of an oval form, in the situation of the left testicle. Fluid was distinguished at the anterior part, and the testicle was to be felt at the posterior part, occupying the same place as in hydrocele.

October 2d.—I punctured the tumor with a lancet. About three ounces of dark-coloured grumous fluid were evacuated; but this did not cause the whole of the tumor to disappear.

The wound made by the lancet did not heal by the first intention. Considerable inflammation and swelling of the scrotum, and skin and cellular membrane of the penis, ensued. A serous, and afterwards a purulent fluid, deeply tinged with blood, was discharged through the opening. By degrees the discharge became less discoloured: on the 15th of October, it consisted of pure pus, and the inflammation of the penis and scrotum had in a great degree abated.

18th.—Pus continued to be discharged through the puncture. A collection of pus was discovered in the lower part of the tunica vaginalis, which did not readily escape through the original opening. A fresh opening was made in this situation, and a good deal of pus escaped.

The abscesses were slow in healing, and it was not until nearly the end of November that the patient was dismissed from the hospital as cured.

Case XXXI. *Hæmatocele, in which Inflammation of the Tunica Vaginalis followed the Puncture of the Tumor, terminating in a Cure without the formation of Abscess.*

Jacob Hill, sixty years of age, was admitted into St. George's Hospital, under the care of Mr. Keate. He had a tumor in the situation of one testicle, of a pyramidal form, having a good deal of the character of a common hydrocele, except that, when a lighted candle was placed behind it, it was found to be perfectly opaque. There was no pain in the tumor, but some pain in the loins.

The patient said that, a year before his admission into the hospital, he had strained himself in lifting a heavy weight, and that the swelling immediately followed this slight accident, but became larger afterwards.

Mr. Keate punctured the tumor with a trocar, and drew off ten ounces of dark-coloured bloody fluid. Little or no solid substance remained after the fluid was evacuated.

The wound made by the trocar healed by the first intention. Nevertheless, inflammation of the tunica vaginalis took place to about the same extent as after the injection of a hydrocele. When this inflammation had subsided, and the swelling dependent on it had disappeared, there were found no traces of the original disease, and the patient was discharged as cured.

Case XXXII. *Hæmatocele, in which the Tunica Vaginalis was several times punctured without Suppuration being induced, and the Patient died of another Disease; with the Appearances on Dissection.*

I saw this patient with my friend and colleague Mr. Jeffreys, through whose kindness I also had the opportunity of examining the appearances on dissection.

A. B—, fifty-seven years of age, in the summer of 1825 received a blow on one testicle, which was followed by a swelling of the size of a goose's egg. This swelling had begun to diminish, when some time afterwards he received another blow, which was followed by an additional swelling. In April 1827, when he came under the care of Mr. Jeffreys, there was a large tumor in the situation of the left testicle, manifestly containing fluid. Mr. Jeffreys punctured the tumor, and several ounces of fluid were drawn off, manifestly blood in a state of dissolution. The tumor, however, did not disappear entirely. Fluid became again collected, and the tumor was again punctured. The operation was repeated twice more; the bloody appearance of the fluid drawn off each time being less distinct than it was the time before.

In the beginning of June, the patient was unfortunately seized with erysipelas of the head, attended with some very severe symptoms, and of this disease he died on the 15th of June.

On dissection, the tunica vaginalis was found much thickened, and it contained nearly an ounce of bloody serum. The internal surface of the tunica vaginalis was irregular, and had portions of what appeared to have been coagulum of blood adhering to it. Towards the upper and outer part of the testicle, a pendulous pyriform tumor, of the size of a hazel-nut, was attached by a narrow neck to the inner surface of the tunica vaginalis. The tumor was covered externally by a smooth (and apparently secreting) membrane; but internally it consisted of a mass of coagulated blood, not very unlike that which is found in the sac of an aneurism.

II. Hæmatocele occurs not unfrequently in combination with hydrocele, a circumstance easy to be accounted for when we consider that, in cases of this last disease, the increased bulk of the parts must render them particularly liable to suffer from accidental injury.

In the majority of cases only a small quantity of blood is blended with the fluid of the hydrocele, and a cure is effected by a very simple process. The tumor may be punctured with a trocar, and the fluid drawn off in the usual manner. This operation may be repeated whenever the fluid is again accumulated, until the whole of the blood has become dissolved, and the red tinge in the fluid drawn off has disappeared; after which port-wine and water, or some other stimulating fluid, may be injected, as in ordinary cases of hydrocele.

In other cases, however, where a large extravasation of blood has taken place, a cure by the method just described is either very tedious or absolutely impracticable, and it is necessary to resort to a more serious operation.

CASE XXXIII. *Hæmatocele existing in combination with Hydrocele; both Diseases being cured by the Incision of the Tunica Vaginalis.*

James Webb, thirty years of age, admitted into St. George's Hospital July 17th, 1824. Nearly two years before his admission, he first observed a swelling, unattended by pain, in the situation of the left testicle. The swelling was at first trifling, but gradually increased in size, giving no inconvenience except what arose from its bulk, and having all the characters of common hydrocele.

A week before his admission into the hospital, while making an unusual exertion in loading hay, he experienced a sensation as if something had burst into the scrotum, and the tumor became suddenly very much increased in size. In the evening of this day, he was seen by Mr. ROSE, of High Wycombe, who directed the application of a spirituous lotion. Two or three days afterwards, Mr. Rose made a puncture with a trocar, and drew off a considerable quantity of bloody fluid. The tumor, however, still remained of a large size.

At the time of the man being admitted into the hospital, there was a tumor in the situation of the left testicle, of an oval form, but somewhat smaller above than below. Measured from above downwards, the length of it was about eighteen inches, and its transverse diameter was about five or six inches. In most parts the fluctuation of fluid was distinctly perceptible, but in other parts it seemed to consist of solid substance. The spermatic cord was free from disease, and the general health was unaffected.

On the 23d of July, I made a longitudinal incision, several inches in length, into the tunica vaginalis. It was found distended with a dark-coloured bloody fluid, having fragments of coagulum floating in it. When the contents of the tunica vaginalis had escaped, the membrane itself was found to be thickened, and the inner surface of it was every where encrusted with layers of coagulum, at the same time bearing the marks of considerable inflammation. A good deal of the cellular membrane of the scrotum was in a sloughy state. The testicle itself was seen in its natural situation, and apparently healthy.

A portion of the skin and tunica vaginalis were removed with the knife, and the parts were dressed with dry lint in contact with the tunica vaginalis.

No unfavourable symptoms followed the operation. Suppuration took place of the inner surface of the tunica vaginalis ; the parts contracted; and in a few weeks the patient left the hospital as cured.

Case XXXIV. *Hæmatocele combined with Hydrocele; both Diseases cured by the Incision of the Tunica Vaginalis.*

James Mason, fifty years of age, admitted into St. George's Hospital, July 19th, 1826. There was a tumor, of the size of a large melon, in the situation of the right testicle. The tumor was ponderous, opaque, evidently containing fluid, though the fluctuation was less distinct than in a common hydrocele. The testicle was perceptible at the posterior part, where pressure gave to the patient the peculiar sensation which arises from squeezing the testicle under ordinary circumstances.

He said that, in the summer of 1821, he first perceived a swelling connected with the right testicle, which gradually increased, without pain or tenderness. In May 1823, a surgeon introduced a trocar, and drew off a considerable quantity of transparent fluid. In a short time after the puncture, the tumor returned, and increased gradually as before. On the 14th of May last, he was carried to bed in a state of intoxication. He had no recollection of having received a blow, though this might well have happened without his being conscious of it. When he awoke in the morning of the 15th of May, he found the tumor increased to an enormous size, and the scrotum of a livid colour. His attention was called to it, not by pain, but by the sudden addition to the bulk of the scrotum. After this, instead of increasing further, the tumor gradually and slowly diminished up to the time of the man being admitted into the hospital.

On the 21st of July, I made a free incision into the anterior part of the tunica vaginalis. A large quantity of bloody serum immediately escaped, and numerous masses of coagulum were then removed with the fingers. The tunica vaginalis having been emptied, the testicle was discovered, apparently healthy, at the posterior part. Lint was placed between the edges of the wound, with some simple dressings over it, and the patient was directed to remain in bed.

July 22d and 23d.—He was going on well.

24th.—In the evening, he was seized with a rigor, followed by heat and thirst, and increased frequency of pulse.

On the following day, the pulse was frequent, the skin hot and dry, the tongue dry, and inclined to a brown colour; the scrotum was inflamed and swollen. The inner surface of the tunica vaginalis was in a state of suppuration.

The inflammation of the scrotum extended to the skin and cellular texture of the penis, and was attended with a good deal of febrile excitement of the system. In about a week these symptoms began to subside.

An abscess, however, formed in the cellular texture at the posterior part of the scrotum, which was opened on the 11th of

August. This abscess healed readily. The cavity of the tunica vaginalis became gradually obliterated by granulations; and, on the 6th of September, the patient left the hospital, at which time the scrotum was reduced to nearly its natural size, and there were no marks of disease except a small superficial ulcer, which healed in a few days afterwards.

(To be continued.)

---

